# Layered Double Hydroxides as Rising-Star Adsorbents for Water Purification: A Brief Discussion

**DOI:** 10.3390/molecules27154900

**Published:** 2022-07-31

**Authors:** Brígida Maria Villar da Gama, Rangabhashiyam Selvasembian, Dimitrios A. Giannakoudakis, Konstantinos S. Triantafyllidis, Gordon McKay, Lucas Meili

**Affiliations:** 1 Laboratory of Processes, Center of Technology, Federal University of Alagoas, Maceió 57072-900, AL, Brazil; brigidavillar@gmail.com; 2 Department of Biotechnology, School of Chemical and Biotechnology, SASTRA Deemed University, Thanjavur 613401, Tamilnadu, India; rambhashiyam@gmail.com; 3 Department of Chemistry, Aristotle University of Thessaloniki, 54124 Thessaloniki, Greece; ktrianta@chem.auth.gr; 4 Division of Sustainable Development, College of Science and Engineering, Hamad Bin Khalifa University, Qatar Foundation, P.O. Box 5825 Doha, Qatar; gmckay@hbku.edu.qa

**Keywords:** layered double hydroxides (LDHs), removal, pollutants, wastewater treatment

## Abstract

Within the frame of this article, briefly but comprehensively, we present the existing knowledge, perspectives, and challenges for the utilization of Layered Double Hydroxides (LDHs) as adsorbents against a plethora of pollutants in aquatic matrixes. The use of LDHs as adsorbents was established by considering their significant physicochemical features, including their textural, structural, morphological, and chemical composition, as well as their method of synthesis, followed by their advantages and disadvantages as remediation media. The utilization of LDHs towards the adsorptive removal of dyes, metals, oxyanions, and emerging pollutants is critically reviewed, while all the reported kinds of interactions that gather the removal are collectively presented. Finally, future perspectives on the topic are discussed. It is expected that this discussion will encourage researchers in the area to seek new ideas for the design, development, and applications of novel LDHs-based nanomaterials as selective adsorbents, and hence to further explore the potential of their utilization also for analytic approaches to detect and monitor various pollutants.

## 1. General Background

Freshwater quality has been threatened over the few last decades due to rapid urbanization. To meet the enhanced demand for products, the expansion of industrial and agricultural activities is taking place at an accelerated pace, which ends up releasing a large number of contaminants into water bodies [1,2]. In turn, these activities can lead to water contamination with various types of chemical substances, which are constantly and well reported in the specialized literature; namely dyes, metals, refining substances, pharmaceuticals, fertilizers, personal care products, pathogenic bacteria, and more. The most common between of them are:

Dyes: Various colored substances are widely utilized as dyes by the textile, food, paper, and pharmaceutical industries. Among these sectors, the textile industry releases 10 to 15% of the dyes produced in the environment. These elements, even in low concentrations, can pose a threat to the environment and living beings. The presence of dyes in water resources reduces the penetration of light through the water, affecting photosynthesis and dissolved oxygen levels, and harming the aquatic biota. In addition, some dyes can degrade to produce highly toxic and carcinogenic compounds [3,4,5].

Metals: Effluents from industries such as battery manufacturing, electroplating, mining, smelting, and many other industrial activities consist of heavy metals mixtures (such as Cd, Pb, Hg, Cu, Ni, Cr, As). The presence of these metals in water bodies is harmful to human health, as they are bioaccumulative and toxic. Among the negative effects of these elements in living beings, even at a low concentration are diarrhea, stomach pain, headaches, chronic bronchitis, and lung cancer [3,6,7].

Pharmaceuticals: Drugs are widely used in human medicines, livestock, aquaculture, beekeeping, and poultry to aid species growth. Currently, the release of these elements into the environment is not always absolutely regulated/controlled; hence, it is difficult to monitor their effects on living beings. The discharge of drugs into water resources occurs through the excretion of compounds not metabolized by living beings and industrial and hospital effluents. The continued presence of these pharmaceutical pollutants can cause oxidative stress and negative effects on reproduction (sperm motility and abnormal fetal development), osmoregulation, and altered immune functions in aquatic biotics [8,9].

Emerging pollutants: In general, emerging pollutants are chemical or microbiological products that are not commonly monitored or regulated and can cause problems to the environment as well as to the health of living beings. Except for the abovementioned, substances that can end up acting as pollutants are usually found in personal care products, pharmaceuticals, industrial additives, pesticides, plasticizers, and solvents. Among these substances, exposure to emerging organic compounds such as phenol, benzene, toluene, and xylenes can cause serious health problems such as gastrointestinal disorders, lung and kidney damage, heart attacks, and cancer. Yet, the increased use of pesticides and personal care products has increased dramatically in recent years. Although these products appear in low concentrations in water bodies, they can negatively influence the environment due to their resistance to biodegradation and accumulation in tissues [3,10].

Among the several technologies applied for water treatment, adsorption using layered double hydroxides (LDHs) will be highlighted in this article. Adsorption is a unitary operation in which the separation process occurs due to contact between a fluid phase—in this case, liquid—containing one or more contaminants (adsorbate) to be adsorbed/removed, and a solid (adsorbent). Due to the imbalance of forces (attraction or repulsion) for adsorption, the contaminant is attracted to the solid surface by physical or chemical interactions. This mass transfer process occurs until the balance between the adsorbed contaminant and what remains in the liquid phase, the residual, is reached. This is termed equilibrium. Adsorption is recognized as an effective process for water treatment due to certain characteristics, such as high efficiency, feasibility, low cost, flexibility, simplicity, wide processing range, cost-effective applications for water treatment, easy operation and implementation, great availability, and the possibility of adsorbents’ regeneration. A very important aspect is that the adsorption-based processes do not result in the formation of hazardous substances/by-products [1,3,11,12,13].

Around 70% of the adsorption operation cost is related to the adsorbents [14]. Therefore, scientists involved in this area play a fundamental role in the development, characterization, and optimization of novel adsorbents. Hence, we seek materials with low cost and ease of production, high efficiency and selectivity towards the removal of the targeted contaminant, large surface area and volume of accessible pores, and good mechanical resistance. It is also desirable for the adsorbents to be regenerated/reused for numerous sufficient cycles to justify the costs.

Currently, and within this context, layered double hydroxides (LDHs), also known as anionic clays, have drawn great attention in their application as adsorbents. Several researchers have proposed the use of LDHs as efficient adsorbents against a plethora of organic and inorganic contaminants [4,15,16,17,18]. Furthermore, recent research demonstrates that biochar/LDH co-blends are highly promising, sustainable, and eco-economic materials for water treatment. Conventional adsorbents such as activated carbon have specific limitations such as high cost, low reuse performance, and low selectivity for water treatment. On the other hand, biochar/LDH blends can have a low cost, high surface area, an elevated amount of active adsorption sites, inherent interchangeability, a considerable increase in anions, and less toxicity for the removal of organic substances [19,20,21,22,23]. A wide variety of works have focused on evaluating the adsorptive capacity of LDHs against various pollutants such as dyes, drugs, arsenic, rare earth, radioactive substances, phosphate, metals, among other pollutants. Table 1 collects the most important advantages and disadvantages of using LDHs for water treatment compared to other materials [19,20,21,22,23,24,25,26].

**Figure 1:**
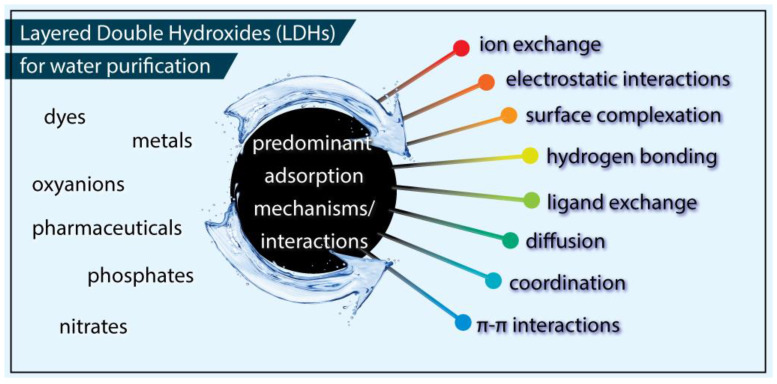
All the reported interactions/mechanisms involved in the removal of various pollutants by Layered Double Hydroxides (LDHs).

Considering the enhanced surface chemical heterogeneity and the nanostructured nature of LDHs, their elevated remediation efficiency against different substances is linked in the literature to plenty of physical and/or chemical interactions, with the most reported ones collected in Figure 1 [15,27]. Among all the interactions/mechanisms involved in the removal of various pollutants by Layered Double Hydroxides (LDHs), the most predominantly reported are:

(1) Physical adsorption. LDHs can have a high specific surface area, and hence present high adsorption capacities due to the presence and availability of active adsorption sites. Furthermore, the specific surface area of LDHs can be increased through calcinating or modifying/depositing on supports with three-dimensional structures.
(2) Ion exchange. Strongly negative molecules can be easily changed for the original anions in LDHs. In addition, positive ions can also be exchanged in the intermediate layer of LDHs, if pre-interleaved by some chelators.
(3) Interleaving. This starts from a preparation process, such as co-precipitation. Furthermore, the capture of molecules via the intercalation process is faster and more complete than ion exchange.

## 2. LDHs Physicochemical Characteristics

For the design and utilization of a proper adsorbent, certain characteristics are essential, such as low production cost, thermal, mechanical, and chemical stabilities, desirable physicochemical characteristics (such as elevated textural properties and high surface functional groups availability), high efficiency and adsorption capacity, rapid kinetics, and regeneration/reuse potential [28]. Some of the mentioned properties can only be verified when applying the material in a specific process due to the dependence on operational conditions and adsorbate characteristics. Of course, it is very challenging to develop materials that possess all the abovementioned characteristics. Thus, scientists involved in the water purification area of research have developed and tested a broad range of materials. Among them, layered double hydroxides (LDHs) are prominent and meet many demands, considering the sustainability of the approach, high anion exchange capacity, high specific surface area, high ion exchange capacities, and regenerative adsorptivity [28]. In addition to their attractive properties as adsorbents, LDHs have applications in various fields such as drug carriers, catalysis, flame retardants, pharmaceutical transport systems, electrocatalytic water separation, additives for polymers, photocatalytic degradation, and medicine [6,18,29].

**Figure 2:**
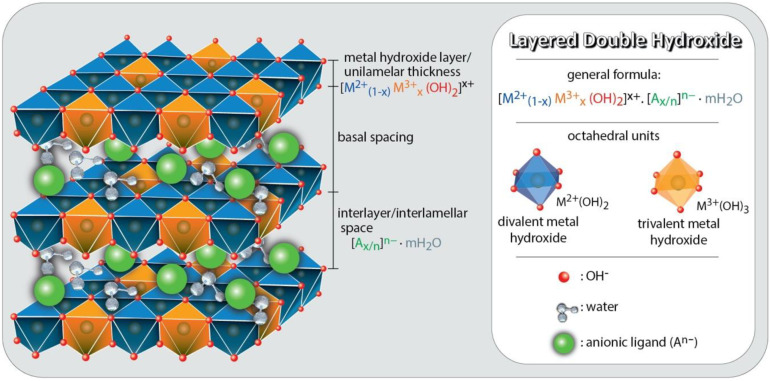
A schematic presentation of the layered double hydroxides (LDHs) chemical composition and structure. Figure reproduced from reference [30]. Copyrights Elsevier, 2022.

LDHs are two-dimensional (2D) nanostructures composed of stacked layers consisting of mixed hydroxides of di- and trivalent cations with hydrated anions in the spaces between the positively charged *lamellae* (Figure 2). LDHs are also called hydrotalcite compounds, due to the rigid layer structure derived from brucite with edge-sharing M(OH)_6_, similar to flexible graphene oxide nanosheets [30]. In general, such materials are represented by the formula: [M^2+^_1−x_M^3+^_x_(OH)_2_]^x+^. A^m−^_x/m_.nH_2_O [30], where M^2+^ represents a divalent metal cation; M^3+^, the trivalent cation; A^m−^, an anion intercalated with charge m; X, the ratio between divalent (M^2+^) and trivalent cations (M^3+^), for which the value of the molar ratio can be between 2.0 and 4.0; and *n*, the number of soft water molecules. Many divalent (such as Mg^2+^, Fe^2+^, Ca^2+^, Co^2+^, Cu^2+^, Ni^2+^, and Zn^2+^) and trivalent (such as Al^3+^, Cr^3+^, Ga^3+^, Mn^3+^, and Fe^3+^) metal cations can be used for the preparation of LDHs. Except for bivalent and trivalent metal ions, a wide range of monovalent and tetravalent metal ions (such as Li^+^, Ti^4+^, Sn^4+^, or Zr^4+^) may also be inserted in the octahedral sites [31]. In addition, the electroneutrality of LDHs can be due to the presence of hydrated organic or inorganic anions (${\mathrm{CO}}_{3}^{2-}$, ${\mathrm{NO}}_{3}^{-}$, ${\mathrm{SO}}_{4}^{2-}$, OH^−^, Cl^−^, Br^−^) in the interlamellar space [18]. Because of these and the different combinations between the cations that participate in the formation of the layers, LDHs can have numerous structural varieties. However, this combination must consider the octahedral coordination and the ionic radius (preferably between 0.50 and 0.74 Å), as distortions may occur with the use of cations. In addition, it is necessary to consider the relationship between the size and charge of the interlayer anion so that it is possible to balance the positive charges of the layer homogeneously [32].

For LDHs, the precisely controlled chemical composition of the lamellar layers and the interlayer composition provide a unique supramolecular nanometric structure with the ability to disperse active sites on an atomic scale, in addition to facilitating morphological manipulation [33,34]. Thus, the versatility of the chemical composition and nanostructure of LDHs establish them as promising adsorbents against a wide variety of pollutants. The most attractive properties of LDHs are the chemical compositions according to the metals used, the space between layers, and the high surface capable of adsorbing bioactive substances, which are dominated by the synthesis conditions [32]. All the above can be tuned on demand based on the synthetic approach/protocol. Another important feature of LDHs is the ease, low cost, and variety of available synthesis methods, which can be classified as direct and indirect methods:

(i) Direct methods: The preparation of LDHs occurs via direct precipitation from the addition of tri- and divalent cations, in a solution in alkaline pH with the main methods of coprecipitation, salt–oxide, sol–gel, induced hydrolysis, and hydrothermal synthesis.
(ii) Indirect methods: involve replacing an interlamellar anion from a previously produced precursor LDH. Examples of this substitution method are ion exchange in solution, ion exchange in acidic medium, double phase replacement, and regeneration through the delaminate precursor [35,36,37]. Therefore, the supramolecular structure, the facile manipulation of adsorption sites at the atomic scale, the versatility of compositions, in addition to the possibility of morphological manipulation create the possibility of tuning the amount and accessibility of the active adsorption sites, and hence the adsorption kinetics, as well as the efficiency for a specifically targeted pollutant [34]. As in any case, LDHs have some specific characteristics that can complicate their use as adsorbents. The low mechanical resistance is a problem for continuous water treatment units and in certain regeneration processes, as LDHs can be exfoliated. Therefore, there is a series of studies in the literature proposing to support LDHs in larger and recalcitrant particles [38,39,40]. In addition, in acidic media, the removal capacity of LDHs is compromised due to low structural stability at low pH [26]. In Table 2, we collected characteristics of methods of synthesis which can be followed for the preparation of pure LDHs, as well as their composites and hybrids [18,25,41,42].

## 3. LDHs as Adsorbents

As already mentioned, LDHs are very promising materials for the removal of a wide variety of pollutants via adsorption. There are many reports in the literature; consequently, we will address some of the most important ones that gather the highest level of research attention for specific pollutants, with emphasis on the utilized LDHs.

Dyes: The presence of dyes in water and wastewater is highly undesirable, and LDHs have been reported as efficient remediation media of a large number of industrial dyes [4]. Dyes are mainly categorized into anionic and cationic dyes. LDHs can remove anionic dyes in the same order of magnitude as cationic dyes in activated carbon [43,44]. Examples of anionic dyes include methyl orange, amaranth, sunset yellow FCF, reactive blue 21, Eriochrome Black T, Congo red, and others. In the literature, it is common to find reports of high adsorption capacities regarding the removal of cationic dyes by LDHs [4,18,45].

El-Abboubi et al. [45] evaluated the double hydroxides of Mg-Al synthesized with dodecyl sulfate (Mg-Al-Ds) and carbonate (Mg-Al-CO_3_), through the coprecipitation method to remove methyl orange from aqueous solution. The prepared LDHs showed the intercalary-like structure with (00l) reflections and a nanoscale nature, as well as the presence of the desired anions in the interlayer space. The maximum capacities were 185.06 mg·g^−1^ for Mg-Al-Ds and 97.5 mg·g^−1^ for Mg-Al-CO_3_. They also observed the influence of the pH of the solution on the adsorption capacity of the dye in the case of Mg-Al-CO_3_. The adsorptive capacity of Mg-Al-Ds was not influenced by the pH of the solution, while Mg-Al-CO_3_ showed greater efficiency at pH in the range of 3–7. According to the authors, this behavior may be associated with two mechanisms: (1) anion exchange of carbonate anions for dye anions; (2) association of the positively charged surface groups of LDHs and dye anions.

Kostić et al. [18] synthesized MgCoAl-CO_3_-LDH double hydroxides using the coprecipitation method for the removal of dye RB19 from an aqueous solution. The material presented a surface area of around 48 m^2^·g^−1^, high crystallinity and the presence of carbonate, hydroxyl, and metal ions groups within its structure. The proposed adsorption mechanisms involved in the process were electrostatic attraction, physisorption, and chemical bonding. The maximum capacity was found to be equal to 367.93 mg·g^−1^.

Ahmed et al. [4] prepared Mg/Fe-LDHs nanoparticles through the precipitation method for adsorption of Congo red dye from effluents. The Mg/Fe-LDHs showed high crystallinity and the presence of hydroxyl functional groups. The adsorption process was governed both by physical and chemical interactions, with the maximum capacity reaching above 9000 mg per gram.

Metals: The discharge of heavy metals into water bodies results in harmful effects to the environment and human health due to their toxicity and persistence. The presence of these elements in water systems has caused concern in recent years; hence, the interest in novel and efficient remediation media is still elevated [9,16]. In the literature, it is quoted that the work of Satoshi Fujji et al. [46], from 1992, was the first in which LDHs were used for metal removal (Pb^2+^, Cu^2+^ and Zn^2+^) [47]. There is no established mechanism for sorption/adsorption of metals in each LDH; hence, the study of the involved mechanism of interaction is still a major challenge for researchers in the field. However, several possible mechanisms/kinds of interactions are proposed, such as surface complexation, isomorphic substitution, surface precipitation, and electrostatic interactions, and chelation with the binding anion has been reported [16,29,48].

Dinari and Neamati [48] synthesized Ca/Fe double hydroxides (LDHs) modified with 3-aminopropyl triethoxysilane as the silane coupling agent. Polyaniline nanocomposites with 5 and 10% by weight of modified silane Ca/Fe LDH-Cit were also studied. Pure polyaniline and that modified with NCs were used as adsorbent to remove Pb^2+^ ions from an aqueous solution. The results showed that NC10% showed higher adsorptive capacity (110 mg·g^−1^; 0.52 mmol·g^−1^) compared to NC5% (56 mg·g^−1^; 0.27 mmol·g^−1^) and NIBP (47 mg·g^−1^; 0.22 mmol·g^−1^), demonstrating the favoring of adsorption in the presence of Ca/Fe double hydroxides.

Guo et al. [29] synthesized ZnNiCr double-layer hydroxides (ZnNiCr-LDHs) using the microwave hydrothermal method to remove Cr^6+^ from an aqueous solution. The material presented a surface area of 354 m^2^·g^−1^, and the SEM analysis showed an irregular block structure. In this study, it was proposed that predominantly electrostatic interaction between the metal ion and the adsorbent took place with a maximum capacity of 28.2 mg·g^−1^ (0.54 mmol·g^−1^). The results demonstrate potential application prospects in the removal of Cr^6+^ in wastewater.

Zhang et al. [16] synthesized sodium alginate intercalated with MgAl-LDH (SA-LDH) for adsorption of Cd^2+^, Pb^2+^, and Cu^2+^ from an aqueous solution. The characterization results showed characteristic peaks at 2θ = 13.24°, 22.88°, 35.09°, 39.19°, 47.23°, and 60.97°, while CO, COO^−^ and CH surface functional groups were also detected in its structure. The maximum adsorption capacities of SA-LDH were 60 mg·g^−1^ (0.945 mmol·g^−1^) for Cu^2+^, 243.66 mg·g^−1^ (1.176 mmol·g^−1^) for Pb^2+^, and 95.55 mg·g^−1^ (0.850 mmol·g^−1^) for Cd^2+^. The results showed that the possible mechanisms involved in the adsorption process are: (1) bonding or complexation with SurOH or Sur-O- of SA-LDH; (2) precipitation of metal hydroxides or carbonates; (3) isomorphic substitution, and (4) chelation with COO^−^ in the interlayers.

Oxyanions: High levels of oxyanions found in the environment are reported all over the world. Oxyanions (e.g., arsenate, chromate, phosphate, selenite, selenate, borate, nitrate, etc.) are considered dangerous to humans and wildlife, even at very low concentrations [17,49]. In the case of oxyanions, the primarily reported mechanisms are ion exchange, electrostatic attraction, and coordination [17,50].

Motandi et al. [51] synthesized zirconium-modified Mg-Al-LDH layered double hydroxides (Zr-LDH) using the coprecipitation method, and then used calcination to obtain an oxide (Zr-LDO), and the materials were used as adsorbents for the removal of phosphate from aqueous solution. The materials showed a well-developed layered structure. In addition, Zr was homogeneously distributed in the adsorbent with a well-defined crystallinity and the presence of brucite, while hydroxyl groups and M–O elongation were detected. The results showed that Zr-LDH and Zr-LDO are excellent adsorbents for phosphate, obtaining maximum adsorption capacities of 99.35 mg/g for Zr-LDH and 80.33 mg·g^−1^ for Zr-LDO.

Jung et al. [17] synthesized Mg-Al double hydroxides (Mg-Al LDHs-FHC) via the one-pot in situ hydrothermal method for the monocomponent and multicomponent adsorption of arsenate and phosphate from aqueous solution. Initially, the influence of the Mg:Al ratio and temperature on the preparation of the adsorbent material was investigated. The results showed that Mg:Al molar ratios and temperature influenced the Mg-Al LDHs-FHC structural properties. The best adsorption efficiency was for Mg:Al molar ratio of 2:1 and temperature of 150 °C. In the one-component system, the maximum adsorption capacities were 56.30 mg·g^−1^ and 33.21 mg·g^−1^ for arsenate and phosphate, respectively. For the multicomponent system, the maximum adsorption capacities were 16.22 mg·g^−1^ for arsenate and 20.26 mg·g^−1^ for phosphate. In the single-component system, a possible adsorption mechanism is the ion exchange between the nitrate interlayer and the arsenate or phosphate groupings. In the multicomponent system, coordinated bonds are also possibly responsible for the competition of arsenate or phosphate in the adsorptive process.

Zhou et al. [50] prepared FeMgMn-LDH via the co-precipitation method for the removal of nitrate from an aqueous solution. The surface area of the material was 47 m^2^·g^−1^; the presence of –OH, N=O, M–OH and M–O–M was detected in its structure. The maximum amount of nitrate adsorption was 10.56 mg·g^−1^. The adsorptive process was spontaneous and exothermic. The possible proposed adsorption mechanisms of nitrate removal using FeMgMn-LDH were an electrostatic attraction and ion exchange.

Emerging pollutants: Plenty of synthetic substances that have recently been detected at low concentrations (ng·L^−1^ or µL^−1^) are assumed to be emerging pollutants nowadays. These components are used in different industrial processes for the production of pharmaceuticals, personal care products (PCPs), beverages, foods, and others [11,12,15,52], and as a result are discarded in aquatic environments. Although LDHs have been reported as efficient adsorbents for various emerging pollutants, such as sodium diclofenac, caffeine, inorganic endocrine disruptor, hormones, and bisphenol A, among others, there are still few studies in the literature.

Santamaría et al. [53] synthesized by the co-precipitation method Zinc–Titanium–Aluminum (ZnTiAl) double layer hydroxides (LDHs) with a Zn/(Al-Ti) molar ratio of 3:1 and studied them for the adsorption of diclofenac and salicylic acid. The study also evaluated the use of commercial aluminum (Al) and aluminum extracted from saline slag. It was shown that the increase in Ti content negatively affected the crystallinity of the material and that the increase in temperature decreased the surface area of the material due to the increase in amorphous mixed oxides. The results showed a great potential of the synthesized hydrotalcites for the adsorption of diclofenac (409 μmol/g) compared to salicylic acid (80 μmol·g^−1^)

Kumari et al. [11] used a double layer of Zn-Al hydroxide (LDH) loaded with Bi_2_O_3_ as an adsorbent for the removal of diclofenac from an aqueous solution. The material presented the main diffraction peaks 003, 006, 012, 041, 111, 241, and 110, and a surface area of 102 m^2^·g^−1^. The Zn-Al-LDH.xBi_2_O_3_ presented a capacity of 574.71 mg·g^−1^ for the removal of diclofenac. The results indicated that the adsorptive process occurs in a monolayer and that the diffusion of diclofenac occurs mainly on the external surface.

Other kinds of pollutants: There are few reports of the use of LDHs for the adsorptive removal of bacteria and viruses, per- and poly-fluoroalkyl substances, and rare earth and radioactive substances; such as Cs, Sr, and Th (excluding Uranium, which is well reported). Therefore, exploring the use of LDHs in the removal of this type of contaminant is very promising and important for environmental health protection.

Table 3 summarizes some cited articles in which LDH were studied as adsorbents against eclectic organic and inorganic substances from aqueous solutions.

Utilization in real-life applications: Industrial effluents contain various pollutants simultaneously, such as dyes, metals, pesticides, and antibiotics, which impose a high physicochemical complexity on the real system. Therefore, it is necessary to understand the interaction between the adsorbates and the adsorbent in the water decontamination process [1,3]. Despite the importance of multicomponent adsorption, there are few references in the literature evaluating the use of LDHs to remove dyes, metals, and rare earth elements in multicomponent systems. Consequently, it is necessary to study the multicomponent adsorption, as well as the evaluation of the selectivity or affinity of a given LDH for each adsorbate and the competition between them. Another developing field is for LDH containing hybrids of two types—nanocomposites and organically modified LDH hybrids—which have been recently reported [54] for the removal of metal ions and dyes from wastewater, with particularly high capacities.

Considering the adsorption mode, batch experiments are well reported in the literature. On the contrary, although the design and efficiency are well approached in lab-scale batch studies, the fixed beds procedure has received little attention regarding exploration of the use of LDHs as adsorbents. The fixed bed columns are applied for continuous flow, and are effective for the treatment of large volumes of effluents, allowing application in industries, adsorption–desorption cycles, efficient fluid–particle contact and easy phase separation, which makes the treatment process cheaper and more sustainable [28,55,56]. In this context, the main challenge is to overcome the LDHs’ poor mechanical resistance for continuous use, because they can be sprayed or exfoliated [40]. In addition, leaching tests should be always considered when studying LDHs in aquatic matrixes.

Fixed beds are employed primarily for commercial water treatment scale-up. Discovering and proposing new operational modes to improve fluid–particle interaction and phase separation is a key issue. Therefore, a smaller treatment area, faster operation, larger water volumes treated, and cost reduction are important advantages. As a result, there may be benefits such as a smaller treatment area, faster operation, treatment of bigger quantities of water, and cheaper costs. Therein, the study of alternative contacting devices, such as fluidized bed, spouted bed, simulated moving bed, expanded bed, continuous stirred tanks, and multi-batch tanks, is much appreciated and warrants substantially further investigation [28].

## 4. Discussion

The studies presented and discussed above demonstrate the prosperity of utilizing LDHs as remediation media, and hence, the growing interest of researchers against various pollutants regarding interpretation and proposals for the involved mechanisms and interactions. The studies revealed elevated adsorptive capacities of organic and inorganic pollutants, even compared to benchmark materials such as porous carbons [4,16,45,48]. The high adsorptive capacity of LDHs is attributed to their high specific surface area, high thermal and chemical stability, but, more importantly, to their surface chemical composition. The characterization of LDHs via various analyses/techniques such as the pH of the point of zero charge (pH_PZC_), Fourier transform infrared spectroscopy (FT-IR), and X-ray diffraction (XRD) helps us to understand the adsorptive mechanism of the process. The most widely proposed interactions/mechanisms involved in the adsorption of organic compounds generally involve electrostatic interaction between the surface of the LDH (positively or negatively charged) with the cationic (positively charged) or anionic (negatively charged) compounds. The adsorptive mechanism associated with the adsorption of metal ions involves the ion exchange between the surface of the material and the metal ions. Other mechanisms are also involved in the adsorption of metal ions in LDHs, such as precipitation and complexation. Among the functional groups present in the structure of LDHs, the hydroxyl group favors the adsorption of both organic and inorganic compounds. Another important parameter in the adsorption process using LDHs is the pH of the solution. It is observed that, in general, the favorable pH for the adsorption of anionic compounds occurs at pH lower than 7 and for cationic compounds at pH above 7.

## 5. Future Prospects

Layered double hydroxides (LDHs) represent one category of materials with an increasing trend of interest toward the removal of water pollutants. To uplift the direction of LDHs-based adsorbent application on a commercial scale, novel approaches and considerations with emphasis on sustainability and low cost are presented. However, more adsorption investigations of LDHs need to be explored in multi-component systems rather than in the current trend, where most of the studies are limited to mono-component systems and lab-scale batch experiments. The parametric influence on the adsorption process requires an advanced optimization approach to attain maximum removal performance of water pollutants using LDHs-based adsorbents. The major setback of a “one time” use of the adsorbent must be overcome with the proper assessment of desorption strategies, followed by a good number of adsorbent recycle options, treated water re-use, and the adsorbate pollutant recovery and re-use. More mechanistic insights into water pollutant removal need to be understood in multicomponent simulated and real effluent systems to study the antagonistic effects targeting the development of adsorbents with high selectivity for the specific pollutant(s). To understand the complete application of layered double hydroxides in water pollutant elimination, the batch system followed by a continuous system needs to be performed at lab and pilot scale, with real effluents.

The simplicity and low cost of LDHs production (for instance, via straightforward coprecipitation of metal salts at mild basic conditions), in addition to the ability to tailor design their composition and structure, establish them as sustainable and attractive candidates for real-life applications. Moving a step forward regarding the advantages of nanomaterials, designing and synthesizing novel nanocomposites/hybrids should be assumed as a worthwhile effort to be further explored in the field of research. The development of synthesis methods capable of producing LDHs on a commercial scale is one of the current problems for the use of these materials on an industrial scale. In addition, LDHs syntheses are generally limited to the use of MgAl and MgFe, requiring the exploration of new compounds. Within the frame of circular (bio)economy and sustainability, the development of novel and advanced composited of LDHs with biomass-derived biochar/carbon and determining the optimum ratio between the counterparts is a prosperous topic of research, since still only a few studies exist.

## Figures and Tables

**Table 1:** Advantages and disadvantages of using LDH in water treatment.

Advantages	Disadvantages
Low cost	Few studies regarding their toxicity in the environment
Sustainable nature	Current methods limit the amount of LDHs produced
Can be engineered for specific purposes	Few studies on its application in real wastewater
Excellent thermal stability	Functional groups preferences for anionic dyes
High removal efficiency	Can be exfoliated during synthesis
Extensive specific surface area	Cannot be easily regenerated/reused
High number of active sites	
Easy to prepare	
Memory effect	
High anion exchange capacities	
Chemical stability	

**Table 2:** Provides details of some of the methods used for the synthesis of LDH.

Methods of Synthesis	Characteristics
Coprecipitation	This method is based on the controlled and slow addition of a base (such as sodium hydroxide and/or bicarbonate, sodium carbonate or ammonium hydroxide) to a solution containing simultaneous divalent and trivalent metal cations. Since more than two cations can precipitate simultaneously, the process must be carried out under supersaturation conditions. It is recommended that the pH of the reaction medium be kept constant in the range of 7–10. Subsequently, the suspension is subjected to hydrothermal treatment to increase the yield or crystallinity.
Salt-oxide	This method was developed by Boehm in 1977 to prepare zinc and chromium LDHs, using an aqueous suspension of ZnO to react with excess CrCl_3_ in an aqueous solution. The salt–oxide method, in short, is a solid–liquid reaction in which the aqueous solution of the excess trivalent ion chloride salt is treated with an aqueous suspension of the divalent metal oxide.
Sol-gel	This synthetic protocol is widely used for the preparation of a plethora of metal oxides due to the possible high efficiency and purity of the final material. One important advantage of this method is the variety of compositions obtained through temperature adjustment. This process consists of the constant agitation of the component that transforms sol to gel. This sol–gel transformation occurs during the strong acid hydrolysis of metallic precursors, predominately using a strong acid such chloric acid or nitric acid. After the formation of the gel, the material is filtered and washed with distilled water, and later with ethanol.
Hydrothermal	The hydrothermal method is generally used when low-affinity anions need to be intercalated into the intermediate layers. This method uses gibbsite and brucite, double-layered hydroxide–deoxycholate intercalation compounds, which are not feasible to obtain easily via other syntheses. An aqueous suspension consists of two oxides, one trivalent metal ion and the other bivalent, which are placed in a pressurized container and subjected to hydrothermal treatment at high temperature for a few days. During this process, the hydrated amorphous precursor crystallizes in the presence of reactive basic oxide.
Ion exchange	This is an indirect method usually applied to pre-synthesized LDHs. This method is used when the anions or the divalent/trivalent metal cations are unstable in the alkaline solution, or when the LDHs have a greater affinity for the guest anions than for the intercalated anions of a pre-synthesized LDH. An aqueous suspension of the LDH precursors/pre-synthesized is mixed with a large excess of the salt of the anion to be intercalated. The reaction is carried out under an inert atmosphere to avoid excess carbonate in the intermediate layers. It is recommended the reaction not occur at pH lower than 4, due to the anion interaction in the LDH layers being weaker and presenting a high temperature in this pH range.
Regeneration/“memory effect”	One of the main properties of LDH is its ability to restructure. After being subjected to heat treatment or calcination (400 to 500 °C), the layered structure of LDH changes to mixed metallic oxides (water, anion, and hydroxyl groups are highlighted). When calcined LDH is placed in a solution containing guest anions, they can recover their original layered structure and form a new LDH phase. This procedure of retrieving its original form (rehydration) is called the “memory effect”, and must be carried out in an inert atmosphere, mostly comprised of nitrogen.

**Table 3:** Some characteristic works in which LDHs are used as adsorbents for organic and inorganic substances.

Pollutant	LDH	Synthesis Method	q_max_ (mg·g^−1^)	Reference
Dye methyl orange	Mg-Al-Ds	Coprecipitation	185.06	[45]
	Mg-Al-CO_3_		97.50	
Dye RB19	MgCoAl-CO_3_-LDH	Coprecipitation	367.93	[18]
Dye Congo red	Mg/Fe-LDHs	Precipitation	9127.08	[4]
Pb^2+^	Ca/Fe LDH-Cit(NC10%)	Precipitation	110.00	[48]
	Ca/Fe LDH-Cit(NC5%)		56.00	
Cr^6+^	ZnNiCr-LDHs	Hydrothermal	28.20	[29]
Cd^2+^	MgAl-LDH (SA-LDH)	Coprecipitation	60.00	[16]
Pb^2+^			243.66	
Cu^2+^			95.55	
Phosphate	Zr-LDH	Coprecipitation	99.35	[51]
	Zr-LDO		80.33	
Arsenate (mono)	Mg-Al LDHs-FHC	Hydrothermal	56.30	[17]
Arsenate (mult)			16.22	
Phosphate (mono)			33.21	
Phosphate (mult)			20.26	
Nitrate	FeMgMn-LDH	Co-precipitation	10.56	[50]
Diclofenac	ZnTiAl	Co-precipitation	0.07	[53]
Salicylic acid			0.01	
Diclofenac	Zn-Al-LDH.xBi_2_O_3_	Hydrothermal	574.71	[11]
